# Impact of Oxygen Supply and Scale Up on *Mycobacterium smegmatis* Cultivation and Mycofactocin Formation

**DOI:** 10.3389/fbioe.2020.593781

**Published:** 2020-12-03

**Authors:** Luis Peña-Ortiz, Ivan Schlembach, Gerald Lackner, Lars Regestein

**Affiliations:** ^1^Junior Research Group Synthetic Microbiology, Leibniz Institute for Natural Product Research and Infection Biology (HKI), Jena, Germany; ^2^Bio Pilot Plant, Leibniz Institute for Natural Product Research and Infection Biology (HKI), Jena, Germany; ^3^Faculty of Biological Sciences, Friedrich-Schiller-University, Jena, Germany

**Keywords:** mycofactocin, redox cofactor, *Mycobacterium smegmatis*, oxygen limitation, glycosylation, ribosomally synthesized and post-translationally modified peptide, tuberculosis

## Abstract

Mycofactocin (MFT) is a recently discovered glycosylated redox cofactor, which has been associated with the detoxification of antibiotics in pathogenic mycobacteria, and, therefore, of potential medical interest. The MFT biosynthetic gene cluster is commonly found in mycobacteria, including *Mycobacterium tuberculosis*, the causative agent of tuberculosis. Since the MFT molecule is highly interesting for basic research and could even serve as a potential drug target, large-scale production of the molecule is highly desired. However, conventional shake flask cultivations failed to produce enough MFT for further biochemical characterization like kinetic studies and structure elucidation, and a more comprehensive study of cultivation parameters is urgently needed. Being a redox cofactor, it can be hypothesized that the oxygen transfer rate (OTR) is a critical parameter for MFT formation. Using the non-pathogenic strain *Mycobacterium smegmatis* mc^2^ 155, shake flask experiments with online measurement of the oxygen uptake and the carbon dioxide formation, were conducted under different levels of oxygen supply. Using liquid chromatography and high-resolution mass spectrometry, a 4–8 times increase of MFT production was identified under oxygen-limited conditions, in both complex and mineral medium. Moreover, the level of oxygen supply modulates not only the overall MFT formation but also the length of the glycosidic chain. Finally, all results were scaled up into a 7 L stirred tank reactor to elucidate the kinetics of MFT formation. Ultimately, this study enables the production of high amounts of these redox cofactors, to perform further investigations into the role and importance of MFTs.

## Introduction

The genus Mycobacterium comprises the highly important etiological agents for human tuberculosis (*Mycobacterium tuberculosis*), bovine tuberculosis (*M. bovis*), and leprosy (*M. leprae*) (Gupta et al., 2018; Parte, 2018). Tuberculosis (TB) is a pulmonary disease, especially relevant for developing countries where multidrug-resistant (MDR-TB) and extensively drug-resistant (XDR-TB) strains are a burden for their development (WHO, 2019). This latter classification is related to the resistance against first-, and second-line antibiotic treatments. Insufficient compliance with the long and cumbersome treatments required facilitates the development of resistance (Nguyen, 2016). *In-vitro* studies on TB pathogenesis, mycobacterial physiology, as well as as the development of novel treatments, are complicated by the high level of biosafety and the low duplication rate of the pathogen, requiring up to 4 weeks to develop visible colonies on agar plates. For this reason, the non-pathogenic strain *Mycolicibacterium smegmatis* mc^2^ 155 (synonym *Mycobacterium smegmatis*) can be a suitable model (Reyrat and Kahn, 2001; Shiloh and Champion, 2010; Yamada et al., 2018).

Mycobacteria involve unusual redox cofactors for the activation or inactivation of some antibiotics. For instance, the reducing agent mycothiol mediates the degradation of antibiotics like rifampicin and isoniazid, whereas Coenzyme F_420_ is involved in the activation of pretomanid (Stover et al., 2000; Rawat et al., 2002; Buchmeier et al., 2003; Singh et al., 2008; Hernick, 2013; Haver et al., 2015). Hence, such compounds arise of medical interest as appealing targets for anti-TB treatments. Therefore, novel redox cofactors present in mycobacteria could open the door to interesting physiological discoveries or even serve as targets for drug development. Mycofactocin (MFT) is a recently identified cofactor, biosynthesized as a ribosomally-produced and post-translationally modified peptide (RiPP) (Ayikpoe et al., 2019; Peña-Ortiz et al., 2020). The discovery of MFT started with the observation that its biosynthetic gene cluster was reminiscent of another bacterial redox cofactor, pyrroloquinoline quinone, and was genomically associated with certain subfamilies of oxidoreductases (Haft, 2011). The MFT locus was found in all species of the genus *Mycobacterium*, is widespread in related Actinobacteria like *Rhodococcus*, but sparsely present in Chloroflexi, Firmicutes, Proteobacteria, and some archaeal species (Haft, 2011; Ayikpoe et al., 2019).

**FIGURE 1:**
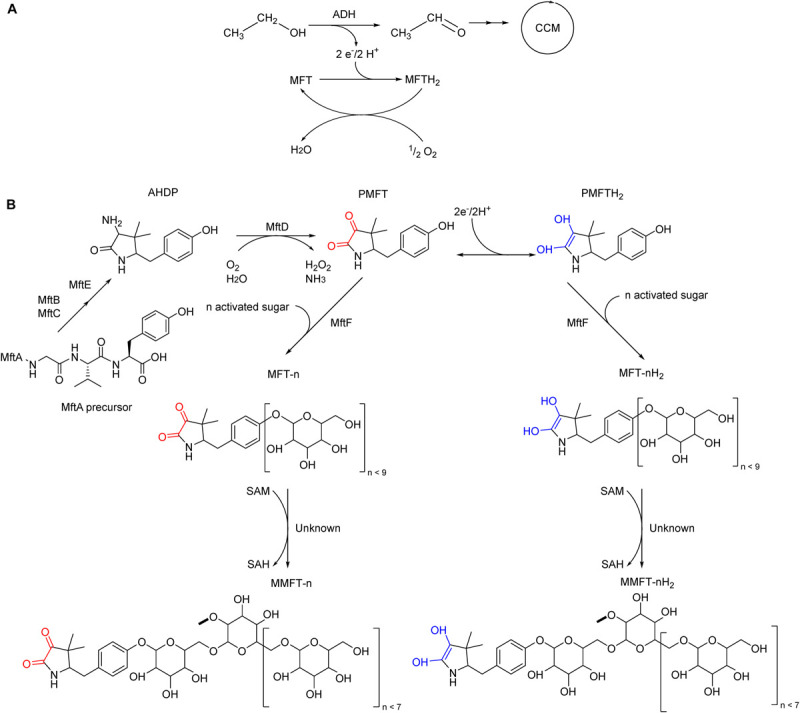
Proposed *in vivo* role of mycofactocin (MFT) and its biosynthesis in *M. smegmatis* mc^2^ 155. **(A)** MFT is the electron acceptor of MFT-dependent alcohol dehydrogenases (ADH), catalyzing the oxidation of ethanol and other primary alcohols to the respective aldehyde for further incorporation into the central carbon metabolism. MFT is reduced to MFTH_2_, which can be oxidized and regenerated by an unknown enzymatic cascade coupled to oxygen as the final electron acceptor. **(B)** Late biosynthetic steps of mycofactocin. The precursor AHDP undergoes oxidative deamination by MftD to generate premycofactocinone (PMFT, redox core red) and premycofactocinol (PMFTH_2_, redox core blue). The glycosyltransferase MftF incorporates up to nine glucose units. A yet-to-be-determined methyltransferase catalyzes the methylation of the second glucose unit.

*In vivo* evidence for MFT serving as a cofactor in the conversion of primary alcohols as sole carbon source into aldehydes for further incorporation into the central carbon metabolism has been found (Figure 1A; Dubey and Jain, 2019; Krishnamoorthy et al., 2019). The cofactor would require regeneration to fulfill this role, which can be accomplished by coupling to additional electron acceptors. Typically, in aerobic organisms, oxygen serves as the final electron acceptor. The enzymes and cofactors mediating MFT regeneration are unknown at present. Pioneering *in vitro* studies described the initial steps of MFT biosynthesis until the formation of a redox-active molecule termed premycofactocin (PMFT) (Bruender and Bandarian, 2016, 2017; Khaliullin et al., 2016, 2017; Ayikpoe et al., 2018; Ayikpoe and Latham, 2019). Recently we reported on the discovery of mature MFT *in vivo* (Figure 1B) and showed that PMFT and PMFTH_2_ are glycosylated with up to nine glucose residues linked by a β-1,4 glycosidic bond. Species with eight residues are the most abundant ones. This decoration is mediated by the glycosyltransferase MftF with activated glucose as the glycosyl donor. To discriminate against the different congeners, the length of the oligoglycoside chain is indicated by the letter n as in MFT-n. For instance, MFT-8 is decorated with eight glucose units. As a redox cofactor, MFT also exists as oxidized and reduced forms. While MFT-n stands for oxidized mycofactocinones, MFT-nH_2_ is used for the corresponding reduced mycofactocinols. To report the sum of oxidized and reduced forms together, the optional hydrogen H_2_ is put in parenthesis, e.g., MFT-8(H_2_). In a previous study, we showed that the second glucose unit exists as 2-*O*-methylglucose (methylmycofactocin, MMFT). MMFT is generally more abundant than non-methylated MFT in *M. smegmatis*. The enzyme responsible for this methylation is not comprised in the MFT gene cluster and is still unknown (Peña-Ortiz et al., 2020), however, it is possible that this modification is catalyzed by a *S*-adenosyl-L-methionine (SAM)-dependent methyltransferase, with *S*-adenosyl-L-homocysteine (SAH) as product. We also detected early products from minor reactions, namely glycosylated AHDP (ADHP-n), methyl glycosylated AHDP (MAHDP-n), and glycyl-containing AHDP (GAHDP). Lastly, we observed strong upregulation of all MFT species during the cultivation of ethanol as carbon source. Both findings are strong support for the hypothesis that MFTs act as a quinone-like hydrogen carrier during ethanol utilization. Further studies on mycofactocins, like enzyme kinetics or crystallization in complex with mycofactocin-dependent oxidoreductases, would require at least milligrams of the pure cofactor (Peña-Ortiz et al., 2020). Chemical synthesis would be a suitable alternative for premycofactocin production, however, synthesis of its glycosylated versions could be hampered by stereoselectivity at the coupling step and the very finely tuned protection and deprotection required (Palcic, 1999; Williams and Galan, 2017).

For the structure elucidation by nuclear magnetic resonance (NMR), preliminary experiments were conducted to produce methylmycofactocins with eight glucose residues, abbreviated as MMFT-8(H_2_) (Peña-Ortiz et al., 2020). However, the generated amounts of all MFTs were too low to enable experiments beyond the structural elucidation. Therefore, systematic development of the bioprocess for a scalable production was essential. Mycobacterium strains are considered strict aerobic or microaerophilic (Moore and James, 1982; Dick et al., 1998; Realini et al., 1998; Lewis and Falkinham, 2015), and the few biotechnological processes available do indicate the importance of oxygen transfer rates (OTRs) in the final product (Hauschild et al., 1994; Lo et al., 2002). Being a redox cofactor involved in aerobic pathways it was reasonable to assume that oxygen availability could have a direct impact on MFT production (Clark and Bushell, 1995; Barberel and Walker, 2000; Gamboa-Suasnavart et al., 2018). In this article, the impact of oxygen supply on production dynamics of MFT from *M. smegmatis* mc^2^ 155 is characterized as well as the scalability of results from shake flask experiments into stirred tank reactors (STR).

## Materials and Methods

### Microbial Strains and Culture Media

*M. smegmatis* was obtained from the Kaufmann laboratory at the Max Planck Institute for Infection Biology in Berlin, Germany. LB complex medium (10 g L^–1^ tryptone, 5 g L^–1^ yeast extract, 10 g L^–1^ NaCl) and Hartman de Bont (Hartmans et al., 1992) mineral medium [2 g L^–1^ (NH4)_2_SO_4_, 0.1 g L^–1^ MgCl_2_⋅6 H_2_O, 3 g L^–1^ Na_2_HPO_4_, 1 g L^–1^ KH_2_PO_4_, 10 mL L^–1^] trace element solution (100 mg L^–1^ EDTA, 20 mg L^–1^ ZnSO_4_⋅7 H_2_O, 10 mg L^–1^ CaCl_2_⋅2 H_2_O, 50 mg L^–1^ FeSO_4_⋅7 H_2_O, 2 mg L^–1^ Na_2_MoO_4_⋅2 H_2_O, 2 mg L^–1^ CuSO_4_⋅5 H_2_O, 4 mg L^–1^ CoCl_2_⋅6 H_2_O, 12 mg L^–1^ MnCl_2_⋅7 H_2_O) were used. In all cases, the production of MFT was stimulated by 10 g L^–1^ of ethanol at the beginning of the production culture.

### Shake Flask Cultivation With Transfer-Rate Online Measurement (TOM)

For the cultivation of the first seed culture, a single colony was used to inoculate 25 mL of LB with 0.05% tyloxapol (Sigma Aldrich), and cultured for 48 h to recover cells in suspension without cell clumps. Then, 25 mL of culture broth was inoculated 24 h at a 10% (v/v) ratio from the first seed. Tyloxapol was not used in the second seed. Both seed cultures were conducted at 37°C and 210 rpm with a shaking diameter of 50 mm.

Afterward, a master mix of LB broth pH 7.3 buffered with 250 mM MOPS was inoculated with 25 mL of second seed culture, 2.5 g of sterile ethanol, and volume was brought up to 250 mL with sterile double-distilled water. For the variation of the OTR and, therefore, the oxygen supply of *M. smegmatis*, four different filling volumes of 10, 20, 30, and 40 mL were chosen in 250 mL non-baffled Erlenmeyer flasks. The maximum filling volume of 40 mL reflects a strongly oxygen-limited condition with a calculated maximum OTR of 12.8 mmol L^–1^ h^–1^ (Meier et al., 2016). The filling volumes of 30, 20, and 10 mL represent the calculated OTRs of 1, 22, and 37 mmol L^–1^ h^–1^, respectively. The lowest filling volume of 10 mL ensures fully oxygen-unlimited conditions. Cultivations were performed in an orbital shaker (TOM,^®^ Kuhner shaker, Switzerland) with a shaking diameter of 25 mm and 210 rpm at 37°C. Every shake flask was equipped with an online monitoring system for oxygen and carbon dioxide measurement. In-house compressed air was supplied at concentrations of CO_2_ and O_2_ of 0.04% (v/v) and 20.95% (v/v), respectively. The low flow setpoint of the mass flow controller was 11 mL min^–1^, while the high flow setpoint was 55 mL min^–1^. Osmolarity was set at 0.5 Osmol L^–1^. Measurements were performed in 10 min cycles with 3 min of measuring time and 40 s of high flow time. Cultures were prepared in duplicates, one duplicate was sacrificed for measurements after a sharp drop (= decrease of at least 30% in 30 min) in OTR was recorded, while the second sample was taken when OTR was 2 mmol L^–1^ h^–1^. Samples were centrifuged; supernatant and pellet were frozen before pH measurement and offline metabolites and MFT measurements as described below. To avoid any impact of evaporation, all measured concentrations are corrected by an evaporation factor. As maximum deviation between duplicate measurements a value below 7% was determined.

### Cultivation in Stirred Tank Bioreactor

Three 300 mL seed cultures were prepared as previously described in 2 L shake flasks, inoculated from a first seed at 10% (v/v) ratio, and cultured for 24 h at 37°C in a 25 mm diameter shaker (Infors HT, Germany). Two were prepared in complex LB medium and one in mineral HdB medium, the latter one supplemented with 10 g L^–1^ ethanol as sole carbon source.

The main bench-scale experiments were conducted in three 7 L stirred-tank reactors (diameter = 160 mm) with a filling volume of 3 L at 37°C. All reactors were equipped with one Rushton turbine (diameter = 64 mm). Two STR were done with complex LB medium under oxygen-limited and unlimited conditions and one STR with mineral HdB medium under oxygen-limited conditions. Each STR was inoculated with the corresponding seed culture at a 10% (v/v) ratio with the same production medium. The aeration rate was constant at 0.75 L min^–1^ (= 0.25 vvm). Under oxygen-unlimited conditions, the dissolved oxygen tension (DOT) was controlled to maintain a minimum level of 20% by the stirring rate at 900 rpm. Under oxygen-limited conditions, the agitation rate was 430 rpm to achieve an OTR of 8 mmol L^–1^ h^–1^. All STR were buffered with 52.3 g L^–1^ (250 mM) MOPS at an initial pH of 7.2 and supplemented with 10 g L^–1^ ethanol, 0.5 g L^–1^ tyloxapol (Sigma Aldrich), and 1 mL L^–1^ antifoam 204 (Sigma Aldrich).

### Analytical Methods

#### Online Measurements in Stirred Tank Bioreactor

For all STR experiments, DOT (Hamilton, United States) and pH were measured online. The O_2_- and CO_2_-concentrations were measured using an off-gas analyzer (Rosemount NGA 2000, Emerson Process Management GmbH&Co., OHG, Haan, Germany) with a paramagnetic sensor and an infrared analyzer. The O_2_-consumption was determined via the OTR, the CO_2_-formation via carbon dioxide transfer rate (CTR), according to the following equations (Anderlei et al., 2004; Regestein et al., 2013):

(1) $$O\,T\,R=\frac{{\dot{V}}_{G}}{V_{L}\cdot V_{n\,o\,r\,m}}\cdot \left(y_{O_{2,i\,n}}-\frac{1-y_{O_{2,i\,n}}-y_{C\,O_{2,i\,n}}}{1-y_{O_{2,o\,u\,t}}-y_{C\,O_{2,o\,u\,t}}}\cdot y_{O_{2,o\,u\,t}}\right)$$

(2) $$C\,T\,R=\frac{{\dot{V}}_{G}}{V_{L}\cdot V_{n\,o\,r\,m}}\cdot \left(y_{C\,O_{2,o\,u\,t}}\cdot \frac{1-y_{O_{2,i\,n}}-y_{C\,O_{2,i\,n}}}{1-y_{O_{2,o\,u\,t}}-y_{C\,O_{2,o\,u\,t}}}-y_{C\,O_{2,i\,n}}\right)$$

The respiratory quotient (RQ) enables first conclusions about the dominance of oxidative or reductive metabolic pathways and is an indicator for metabolic switches during cultivation:

(3) $$R\,Q=\frac{C\,T\,R}{O\,T\,R}$$

#### Offline Metabolite Measurements

Samples of 10 mL were used for metabolites determination. Following harvesting of the cells, supernatant samples were filtered by 0.2 μm and diluted 10-fold with 0.5 mM H_2_SO_4_. A volume of 50 μL was injected in an HPLC system (JASCO International Co., Japan) equipped with a Kromasil 100 C18 (40 mm × 4 mm, 5 μm) precolumn (Dr. Maisch GmbH, Germany) and an Aminex HPX-87H, 300 mm × 7.8 mm, 9 μm ion exclusion column (Bio-Rad, United States), equilibrated at 50°C running in isocratic mode (0.005 mol L^–1^ H_2_SO_4_ at 0.5 mL min^–1^). Detection was done by refractive index detector and UV (215 nm). Metabolite identification was done based on the retention time of known standards. All samples were analyzed at least in duplicates with deviations below 5%.

#### Offline Mycofactocin Extraction and Measurement

For MFT extraction and determination by high-resolution liquid chromatography-mass spectrometry (LC-MS), all solvents used were of LC-MS grade. A sample (duplicates) volume of 10 mL was filtered through a 0.2 μm regenerated cellulose filter (Sartorius), previously washed thrice with 10 mL LC-MS grade water to remove the wetting agent. After applying vacuum, the biomass lawn was washed in the same manner to remove mass spectrometry (MS) interferents like media salts, antifoam, and tyloxapol. The filter was transferred to a 50 mL centrifuge tube containing 20 mL of cold methanol (−20°C) and mixed vigorously on a vortex mixer and an ultrasonic bath for 10 s. The methanolic extract was then transferred to a 100 mL round flask and processed in a rotary evaporator until dryness, then the solids resuspended two times in 500 μL of water and transferred to a microcentrifuge tube. 1 mL of crude extract was centrifuged twice at 17,000 × *g* for 10 min, the supernatant transferred to a new tube and centrifuged again, 600 μL of crude extract was transferred to a glass vial and stored at −20°C. Before LC-MS measurements, all samples were diluted to a 1:10 ratio in LC-MS water.

LC-MS measurements of MFT congeners were performed in an Ultimate 3000 UHPLC coupled to a Q Exactive Plus mass spectrometer equipped with a heated electrospray ionization probe (Thermo Fisher Scientific, Germany) as described before (Peña-Ortiz et al., 2020). Metabolite separation was done in an XB-C18 UHPLC column (150 × 2.1 mm, 2.6 μm, 100 Å, Phenomenex) preceded by a SecurityGuard ULTRA precolumn (2 × 2.1 mm, Phenomenex) at 40°C. 10 μl of the sample was injected and separated chromatographically in a mobile phase composed of 0.1% (v/v) formic acid in either water (A) or acetonitrile (B) and a constant flow rate of 300 μL min^–1^ as follows: 0–2 min, 2% B; 2–15 min 2–99% B; 15–18 min 99% B. Metabolite separation was followed by full scan (MS^1^) in positive ionization mode at two scan ranges: *m/z* 200–600 and *m/z* 580–2,000 at a resolving power of 70,000 at *m/z* 200, injection time to 100 ms, and automatic gain control (AGC) target to 3 × 10^6^. Further details are presented in Supplementary Material 6.

Compound identification by retention time was performed with Compound Discoverer 3.1 (Thermo Fisher Scientific, United States) using the untargeted metabolomics workflow. The default workflow was modified in the Detect Compounds node for a minimum peak intensity of 10,000, and the inclusion of a mass list for targeted identification. The mass list contains molecular mass and retention time of previously identified MFT congeners; maximum RT tolerance for identification was set to 0.25 min. Plotting was done using SigmaPlot 14.0 (Systat Software, Inc., United States). For estimation of MFT production independent of oxidation state, the areas of PMFT (R_*t*_ 7.2) and PMFTH_2_ (R_*t*_ 6.9), MMFT-2 (R_*t*_ 7.1) and MMFT-2bH_2_ (R_*t*_ 7.2), as well as MMFT-8 (R_*t*_ 7.2) and MMFT-8H_2_ (R_*t*_ 6.8) (Peña-Ortiz et al., 2020) were summed up and designated PMFT(H_2_), MMFT-2b(H_2_) as well as MMFT-8(H_2_), respectively. The relative standard error was 31% between duplicates on average for the major mycofactocin species.

## Results and Discussion

### Process Characterization in Shake Flask

**FIGURE 2:**
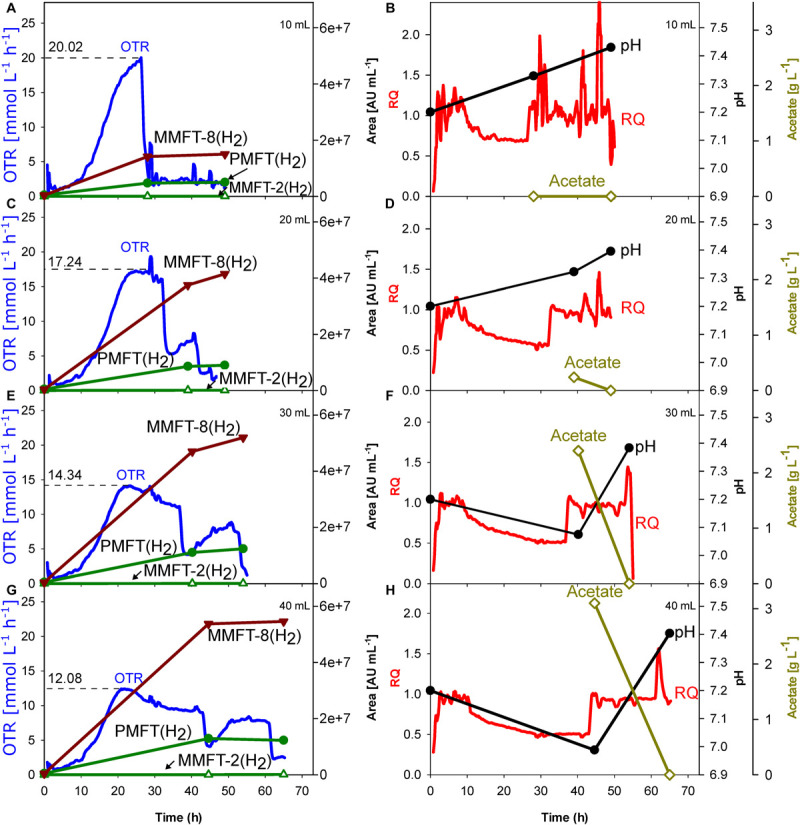
Impact of oxygen supply and limitation on *M. smegmatis* mc^2^ 155 cultivation in shake flask. The oxygen transfer was varied by different filling volumes of the shake flasks. The dotted line reflects the maximum measured oxygen transfer rate (OTR). **(A,B)** 10 mL. **(C,D)** 20 mL. **(E,F)** 30 mL. **(G,H)** 40 mL. Left panels: OTR; PMFT(H_2_), MMFT-2b(H_2_), and MMFT-8(H_2_). Areas under the curve were normalized to sample volume, oxidized and reduced forms were summed up. Right panels: respiratory quotient, pH, acetate. Experimental conditions: Shaking diameter = 25 mm, shaking frequency = 210 rpm, total flask volume = 250 mL, filling volume = 10–40 mL, temperature = 37°C, LB medium with 10 g L^–1^ ethanol, 250 mM MOPS buffer, initial pH = 7.2.

Although oxygen supply is likely a key factor influencing redox cofactor synthesis, most of the published screening experiments with mycobacteria were performed in a shake flask providing no information on oxygen supply. Therefore, the initial experiment aimed to elucidate the impact of oxygen supply on metabolic activity and MFT formation. For this reason, *M. smegmatis* mc^2^ 155 cultivations were performed in a TOM device which enables online measurement of oxygen uptake and carbon dioxide formation (Figure 2). Ethanol (10 g L^–1^) was added as a carbon source to induce MFT production. To determine the impact of decreasing oxygen supply, the filling volume in the shake flasks were increased from 10 to 40 mL, thereby limiting the maximum OTR. The left panels in Figure 2 outline the OTR and offline MFT measurements. Right panels indicate the online RQ, offline pH-value, and acetate formation. Specific characteristic values of all four experimental conditions are presented in Table 1.

**TABLE 1:** Impact of oxygen-(un)limited conditions on metabolic activity and MFT formation of *M. smegmatis* mc^2^ 155 (see Figure 2).

	**Oxygen-unlimited**	**Increasing oxygen limitation**		
Filling volume	10 mL	20 mL	30 mL	40 mL
Calculated OTR_*max*_ [mmol L^–1^ h^–1^]	31.2	18.7	13.5	11
Measured OTR_*max*_ [mmol L^–1^ h^–1^]	20.02 ± 0.08	17.24 ± 0.25	14.34 ± 0.01	12.08 ± 0.47
Duration of oxygen-limited phase (= length of the plateau) [h]	0	10	20	25
Time until ethanol depletion [h]	26.1	31.9	36.5	42.9
Generated acetate_*max*_ [g L^–1^]	0	0.24	2.4	3.1
Premycofactocin_*max*_ PMFT(H_2_) [Area mL^–1^]	6,454,441	10,041,793	13,201,682	12,901,731
Methylmycofactocin-2_*m*__*ax*_ MMFT-2b(H_2_) [Area mL^–1^]	128,868	107,438	100,975	126,746
Methylmycofactocin-8_*m*__*ax*_ MMFT-8(H_2_) [Area mL^–1^]	19,385,519	46,455,040	55,613,267	57,341,055

To ensure oxygen-unlimited cultivation conditions in the shake flask with the lowest filling volume of 10 mL, the theoretical maximum OTR was previously calculated based on an OTR_*max*_-equation published by Meier et al. (2016). The calculated OTR_*max*_ value under these specific conditions is 31.2 mmol L^–1^ h^–1^, representing the theoretical maximum oxygen transfer capacity under the investigated conditions. As the measured oxygen consumption rate never exceeded 20 mmol L^–1^ h^–1^, the oxygen demand by the culture was always well below the calculated maximum oxygen transfer capacity. This is finally validated by the shape of the OTR curve (Figure 2A), which increases until its maximum value without any visible plateau. This statement is also covered by published OTR curves in literature (Anderlei and Büchs, 2001; Büchs, 2001; Maier and Büchs, 2001; Anderlei et al., 2004).

Moreover, no acetate could be detected in the supernatant which is another indicator for unlimited oxygen supply. However, 20 mL filling volume causes a short oxygen limitation phase, which increases up to 25 h with the increasing filling volume. As visible in Figures 2C,E,G the maximum OTR, and thus the metabolic activity decreases from 17 mmol L^–1^ h^–1^ (for 20 mL) to 12 mmol L^–1^ h^–1^ (for 40 mL), prolonging the time until the substrate is consumed. In all cases, an RQ between 0.5 and 0.6 was observed, suggesting that ethanol is the main carbon source in the system (Ramon-Portugal et al., 2004). Simultaneously, acetate production increases with the duration of the oxygen-limited phase (plateau length), from 0.24 g L^–1^ (for 20 mL) to 3.1 g L^–1^ (for 40 mL). The plateau of the OTR curves indicates oxygen-limited conditions. The second peak in the OTR curves is a consequence of the acetate consumption formed before in the oxygen-limited phase. This agrees with a sudden change in the RQ from 0.65 to 1.

Most important is the impact of the different levels of oxygen supply on the formation of different mycofactocins. To increase visibility, only the key (pre-)mycofactocins PMFT(H_2_), MMFT-8(H_2_), and MMFT-2b(H_2_) are depicted in Figure 2, each pool representing the sum of oxidized and reduced forms. The complete overview of all measured mycofactocins and methylmycofactocins including the biosynthetic precursors AHDP-n and the premature cleavage products GAHDP-n, (Peña-Ortiz et al., 2020), can be found in Supplementary Figures S1, S3–S5. The comparison of the four different culture conditions in Figure 2 and Table 1 show clearly that the absolute amount of produced mycofactocins is increased under oxygen-limited conditions, especially the long-chained versions MMFT-7(H_2_) and MMFT-8(H_2_) followed by the aglycons PMFT(H_2_). The reduced molecule is the most prevalent form (see also Supplementary Figure S1). In all cases, the first offline samples were taken when OTR dropped for the first time, and RQ switches from 0.65 to 1, which are both indications for ethanol depletion (Ramon-Portugal et al., 2004). It was assumed that the concentrations of mycofactocins are highest at this time point and could decrease after depletion of the main carbon and energy source ethanol. However, as visible in Figure 2, all MFT concentrations remain constant or even slightly increase when acetate was consumed in the second growth phase.

Since one aim is to investigate and develop a process for generating sufficient amounts of MFT for further experiments, it was decided to scale up the oxygen-limited cultivation conditions of 40 mL filling volume.

### Impact of Scale-Up Into the 7 L Stirred Tank Reactor

As scale-up criteria from shake flask to STR, the volumetric power input was chosen. The aeration rate was adjusted to 0.25 vvm in all cultivations in the STR to reflect the air diffusion through a cotton plug of a shake flask (Maier and Büchs, 2001). The second important parameter for scale-up based on the volumetric power input is the stirring rate (under aerated conditions) which had to be calculated. The power input under shake flask conditions can be calculated using the fundamental equations for the dimensionless Reynolds and modified Power number (Büchs et al., 2000a,b). Calculations for the STR were based on equations published by Möckel et al. (1983). For better readability, all equations, details of geometry, intermediate calculated results, and assumptions are presented in Supplementary Material 1. The calculated value of the stirring rate is 430 rpm, reflecting a power input of 1.48 kW m^–3^ under aerated conditions and should result in combination with an aeration rate of 0.25 vvm in a maximum OTR of approx. 12 mmol L^–1^ h^–1^. The pH was stabilized by the same buffer system and concentration as under shake flask conditions.

**FIGURE 3:**
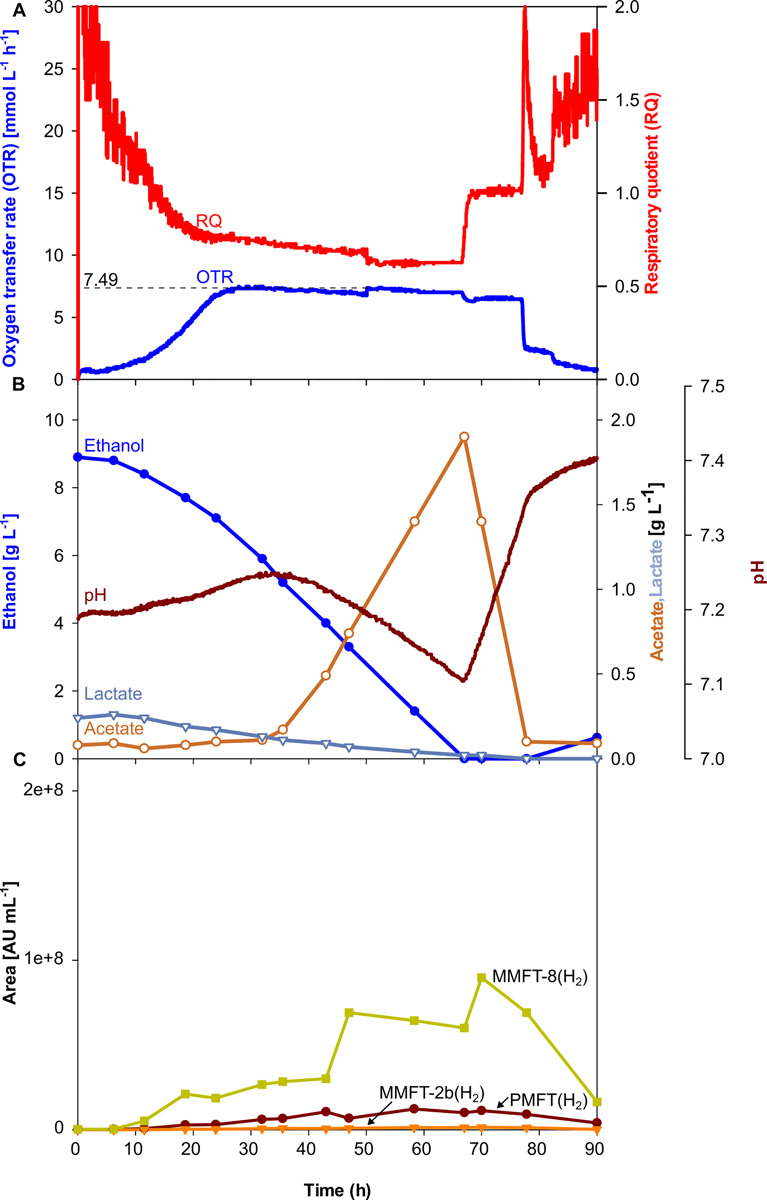
Scale-up of an oxygen-limited batch culture of *M. smegmatis* mc^2^ 155 in a 7 L stirred tank reactor. **(A)** Oxygen transfer rate (OTR) and respiratory quotient. **(B)** pH. Offline metabolites ethanol, acetate, lactate. **(C)** Peak area of selected MFT species by LC-MS. PMFT(H_2_), MMFT-2b(H_2_), MMFT-8(H_2_). Areas were normalized to sample volume, oxidized and reduced forms were summed up. Experimental conditions: Stirring rate = 430 rpm, gas flow rate = 0.75 L min^–1^ (= 0.25 vvm), total reactor volume = 7 L, filling volume = 3 L, temperature = 37°C, LB medium with 10 g L^–1^ ethanol, 250 mM MOPS buffer, initial pH = 7.2.

As visible in Figure 3A, OTR, and RQ follow a trend comparable to the scaled-up shake flask cultivation depicted in Figure 2G. The oxygen was limited at a maximum value of 7.4 mmol L^–1^ h^–1^ which is lower than it was aimed for. The main carbon source ethanol was consumed until its depletion after 66.7 h, indicated by a sharp increase of the RQ-value to 1 (Figures 3A,B). The shake flask experiments had already shown that acetate is formed as an overflow metabolite. The formation of acetate correlates with the beginning of the oxygen-limited growth phase after approx. 28 h, also indicated by a decreasing pH. The previously produced 1.9 g L^–1^ of acetate is taken up in the second growth phase between 66.7 and 77.3 h mirrored by a sharp drop in the OTR curve after its depletion. Based on previous experiences with *M. smegmatis*, biofilm formation was expected and was intended to be controlled by the addition of tyloxapol, a surfactant commonly used in Mycobacterium microbiology to disperse and reduce cell clumping, in combination with antifoam for foaming reduction. Their polymeric nature makes these compounds common LC-MS interferents. The filtration and rinsing strategies were successful in removing these compounds for optimal spectra acquisition, but biofilm and subsequent foaming was still observed, due to submerse aeration and especially on high-density complex medium.

**FIGURE 4:**
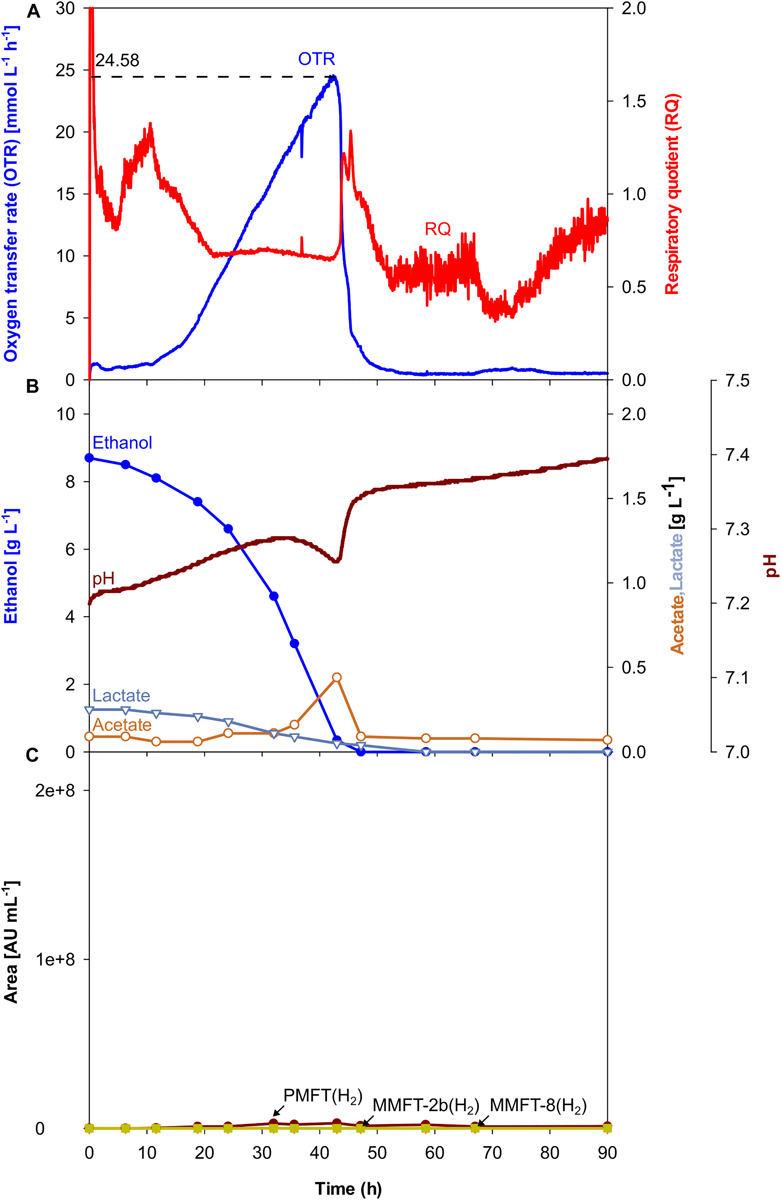
Oxygen-unlimited batch culture of *M. smegmatis* mc^2^ 155 in a 7 L stirred tank reactor. **(A)** Oxygen transfer rate (OTR) and respiratory quotient. **(B)** pH. Offline metabolites ethanol, acetate, lactate. **(C)** peak area of selected MFT species by LC-MS. PMFT(H_2_), MMFT-2b(H_2_), MMFT-8(H_2_). Areas were normalized to sample volume, oxidized and reduced forms were summed up. Experimental conditions: Stirring rate = 900 rpm, gas flow rate = 0.75 L min^–1^ (= 0.25 vvm), total reactor volume = 7 L, filling volume = 3 L, temperature = 37°C, LB medium with 10 g L^–1^ ethanol, 250 mM MOPS buffer, initial pH = 7.2.

Although, all the trends depicted in Figures 2C–H, 3C clearly show that oxygen-limited conditions (after 28 h) have a positive effect on the overall MFT formation, the oxygen-unlimited shake flask experiment presented in Figure 2A contains no information about the kinetics of the different mycofactocins during oxygen-unlimited growth. Therefore, an additional experiment was performed under oxygen-unlimited conditions to elucidate ratios and kinetics. The results are depicted in Figure 4. As visible in Figure 4A, a maximum (unlimited) OTR of 24 mmol L^–1^ h^–1^ was reached at 41.6 h. Only minor acetate concentrations (0.4 g L^–1^, after 43 h) could be detected, which are likely produced by the biofilm at the reactor wall. Ethanol was fully depleted at 47 h (Figure 3B).

The results for MFT formations shown in Figure 4C and Table 2 validate the results of the oxygen-unlimited shake flask experiment (Figure 2A) since only minor amounts of all MFT are formed. Moreover, in comparison to the oxygen-unlimited shake flask experiment, all MFT values are even lower, which can be attributed to the strong foam formation, containing visible amounts of biomass as well as biofilm formation at the reactor wall (Supplementary Figure S6A).

**TABLE 2:** Experimental results of *M. smegmatis* mc^2^ 115 cultures in stirred tank reactors.

	**Increasing oxygen limitation**			
	**Complex medium, oxygen-unlimited (Figure 4)**	**Complex medium, oxygen-limited (Figure 3)**	**Mineral medium, oxygen-limited (Figure 5)**	**Complex medium, strongly oxygen-limited (Supplementary Figure S2)**
OTR_*max*_ [mmol L^–1^ h^–1^]	24	8	8	2.78
Duration of oxygen-limited phase (= length of the plateau) [h]	0	47	28	> 90
Time to ethanol depletion [h]	47.2	67	51.5	N.A.
Generated acetate_*max*_ [g L^–1^]	0.4	1.9	0	0.6
Premycofactocin_*max*_ PMFT(H_2_) [Area mL^–1^]	3,062,618	12,287,516	49,861,224	299,885
Methylmycofactocin-2_*m*__*ax*_ MMFT-2b(H_2_) [Area mL^–1^]	153,306	306,667	2,951,352	744,293
Methylmycofactocin-8_*m*__*ax*_ MMFT-8(H_2_) [Area mL^–1^]	142,371	89,803,844	169,062,399	46,657

To elucidate the opposite effect of extremely low oxygen supply on the MFT formation in complex medium, and STR experiment was performed with a maximum OTR of only 2.5 mmol L^–1^ h^–1^. Due to the negative results concerning the formation of MFT, the details are only presented in Supplementary Figure S2. Ethanol was consumed very slowly, but, most importantly, none of the longer chained (M)MFT-n(H_2_) species were generated in relevant amounts. Only MMFT-2b(H_2_) was produced in relatively high amounts (see Table 2). This experimental result clearly demostrates that an optimal level of oxygen supply is a key parameter to generate MFT but also influences the oligoglycoside chain length of the mycofactocins.

### Impact of Mineral Medium

**FIGURE 5:**
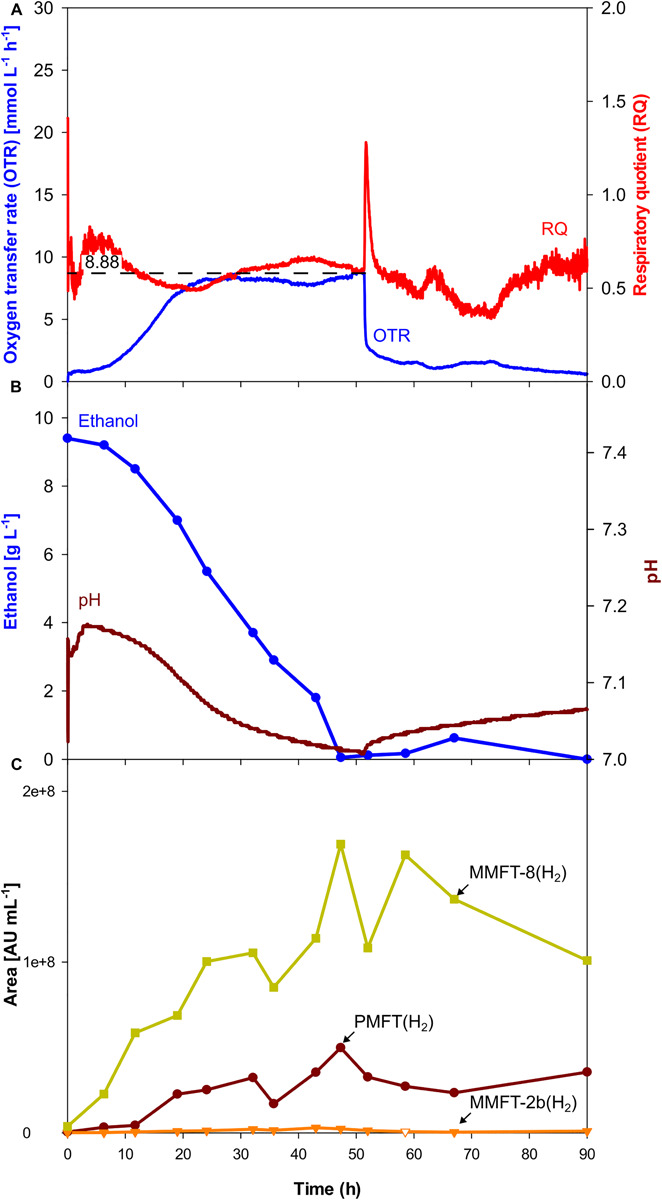
Oxygen-limited batch culture of *M. smegmatis* mc^2^ 155 in a 7 L stirred tank reactor with mineral medium. **(A)** Oxygen transfer rate (OTR) and respiratory quotient. **(B)** pH. Offline metabolites ethanol, acetate, lactate. Panel **(C)** peak area of selected MFT species by LC-MS. PMFT(H_2_), MMFT-2b(H_2_), MMFT-8(H_2_). Areas were normalized to sample volume, oxidized and reduced forms were summed up. Experimental conditions: Stirring rate = 430 rpm, gas flow rate = 0.75 l min^–1^ (= 0.25 vvm), total reactor volume = 7 L, filling volume = 3 L, temperature = 37°C, mineral medium with 10 g L^–1^ ethanol, 250 mM MOPS buffer, initial pH = 7.2.

For further characterization of MFT formation, clearer analytical results, and easier purification, a chemically defined mineral medium Hartmann de Bont (HdB) was tested for further process development as it was used before in several studies involving mycobacteria (Hartmans et al., 1992; Hauschild et al., 1994; Song and Niederweis, 2011; Berney et al., 2012; Greening et al., 2014; Peña-Ortiz et al., 2020). Since previous experiments have proven the positive impact of oxygen limitation, oxygen-limited conditions were also chosen for the experiment presented in Figure 5. As depicted in Figure 5A the OTR increased up to a maximum value of approx. 8 mmol L^–1^ h^–1^ comparable to the previous oxygen-limited cultivation in complex medium (see Figure 3). With a total duration of 28 h, the oxygen-limited phase is shorter in mineral medium comparing to complex medium (see Table 2), since ethanol is the only available carbon source in mineral medium. Moreover, as indicated by the OTR curve, there is only one growth phase without any acetate formation and therefore no second growth phase. This result is validated by the HPLC measurements, which did not detect any organic acids in the supernatants. As before, a sharp drop in the OTR curve indicates the depletion of ethanol at 51.5 h, mirrored by the HPLC results depicted in Figure 5B.

As visible in Figure 5C, the kinetics of MFT formation looks similar to the results of the oxygen-limited cultivation in complex medium (Figure 3). However, as depicted in Table 2, the maximum amounts of all MFT species are doubled, which makes the oxygen-limited cultivation in the mineral medium the most promising conditions for further MFT production.

### Comparison of Mycofactocin Formation

The previously shown experiments have demonstrated that the positive effect of oxygen-limited conditions can be scaled up into the STR and even higher amounts of MFT could be produced under STR conditions. Besides the maximum OTR, the duration of the oxygen-limited phase is an essential parameter to influence not only the overall MFT formation but also the glycosylation of the different MFT species. As shown in Table 2, the effect of a longer oxygen-limited phase is visible but has to be seen in the context of the maximum OTR. The current results suggest elongating the phase of oxygen-limited conditions, which could be realized by higher initial concentrations of ethanol, by the addition of ethanol during cultivation (pulsed-batch) as well as by implementing a fed-batch strategy. In every case, the oxygen supply reflected by the OTR should be adjusted between 8 and 12 mmol L^–1^ h^–1^. The optimal time point for harvesting seems to be right before ethanol is fully depleted, since especially MMFT-8(H_2_) concentration started to fluctuate during the metabolic switch from ethanol to acetate. Depending on the chosen level of oxygen supply, the length of the glycosyl chain can be influenced. As stated in Table 2 the purest amounts of the short-chained MMFT-2b(H_2_) can be generated for levels of very low oxygen supply, realized by OTRs smaller than 8 mmol L^–1^h^–1^. If non-glycosylated PMFT is of interest, mineral medium combined with oxygen-limited conditions will be the most promising conditions.

## Conclusion

This study has shown how the formation of different species of MFT is influenced by cultivating *M. smegmatis* under different levels of oxygen supply. On shake flask level, it could be demonstrated that oxygen-limited conditions result in a four times higher amount of longer-chained MFT species and unexpectedly, even promoted drastic changes in the length of the glycosyl chain of MFT. This finding can be used to direct the biosynthesis toward short or elongated MFT species. After scale-up in a 7 L STR, the detected amount of longer-chained MFT species was further increased and even doubled by changing from complex to mineral medium. In addition, the time point of harvesting was identified as another critical parameter since all MFT was degraded after depletion of the carbon sources. A critical open issue is the current need to add antifoam agents in case of submerged aeration to reduce the foam formation. With respect to the overall process to generate pure mycofactocins on a larger scale, antifoam agents are very difficult to remove in a downstream procedure. Therefore, alternative process strategies have to be applied, like pressure fermentation with strongly reduced aeration, to avoid the addition of antifoam agents. Overall, this study represents a milestone for future large-scale production on milligram- to gram-level of MFT congeners and thus sets the stage for upcoming biochemical investigations as well as for more detailed studies into the biosynthesis and physiology of the cofactor.

## Data Availability Statement

The raw data supporting the conclusions of this article will be made available by the authors, without undue reservation.

## Author Contributions

LP-O and IS conducted the experiments. GL and LR supervised the study. All authors have written the manuscript under the supervision of LR.

## Conflict of Interest

The authors declare that the research was conducted in the absence of any commercial or financial relationships that could be construed as a potential conflict of interest.

## Supplementary Material

Supplementary Material 1 Calculations for scale-up.

Supplementary Material 2 Supplementary Figure S1. All mycofactocin congeners observed in shake flask cultivations.

Supplementary Material 3 Supplementary Figure S2. Strongly oxygen-limited batch culture of M. smegmatis mc2 155 in a 7 L stirred tank reactor.

Supplementary Material 4 Supplementary Figure S3. All mycofactocin congeners observed in complex medium under oxygen-limited conditions. Supplementary Figure S4. All mycofactocin congeners observed in complex medium under oxygen-unlimited conditions. Supplementary Figure S5. All mycofactocin congeners observed in mineral medium under oxygen-limited conditions.

Supplementary Material 5 Supplementary Figure S6. Foam formation and growth at the reactor wall.

Supplementary Material 6 Additional information for LC-MS analysis.

## Abbreviations

AGC: Automatic gain control
CTR: Carbon dioxide transfer rate
DOT: Dissolved oxygen tension
LB: Lysogeny Broth
LC-MS: Liquid chromatography–mass spectrometry
MFT: Mycofactocin
MMFT-2b: Methylmycofactocin containing 2 glucose moieties (oxidized)
MMFT-2bH_2_: Methylmycofactocin containing 2 glucose moieties (reduced)
MMFT-2b(H_2_): Methylmycofactocin containing 2 glucose moieties (reduced and oxidized)
MMFT-8: Methylmycofactocin containing 8 glucose moieties (oxidized)
MMFT-8H_2_: Methylmycofactocin containing 8 glucose moieties (reduced)
MMFT-8(H_2_): Methylmycofactocin containing 8 glucose moieties (reduced and oxidized)
OTR: Oxygen transfer rate
PMFT: Premycofactocinone (oxidized)
PMFTH_2_: Premycofactocinol (reduced)
PMFT(H_2_): Premycofactocin (reduced and oxidized)
RQ: Respiratory quotient
STR: Stirred tank reactor
${\dot{V}}_{G}$: Gas flow rate [L h^–1^]
*V*_*L*_: Filling volume of shake flask or STR [L]
*V*_*norm*_: Molar volume of an ideal gas [mol L^–1^]
*y*_*CO_2,in*_: Molar fraction of carbon dioxide in the gas inlet
*y*_*CO_2,out*_: Molar fraction of carbon dioxide in the off gas
*y*_*O_2,in*_: Molar fraction of oxygen in the gas inlet
*y*_*O_2,out*_: Molar fraction of oxygen in the off gas.
