# Supplementary material for: Impact of Oxygen Supply and Scale Up on Mycobacterium smegmatis Cultivation and Mycofactocin Formation
Source: Front Bioeng Biotechnol. 2020 Dec 3;8:593781. doi: 10.3389/fbioe.2020.593781 (PMC7744413; doi:10.3389/fbioe.2020.593781)
Supplement: Supplementary Material 1 — Calculations for scale-up. [file Data_Sheet_1.PDF]

Supplementary Material for "Impact of oxygen supply and scale up on Mycobacterium smegmatis *cultivation and mycofactocin formation"*

1 Calculations for scale up

$$Re = \frac{\rho \cdot n_{flask} \cdot d_{flask}}{\eta} \quad (4)$$

$$Po'_{flask} = 70 \cdot Re^{-1} + 25 \cdot Re^{-0.6} + 1.5 \cdot Re^{-0.2} \quad (5)$$

$$Po'_{flask} = \frac{P_{flask,aerated}}{\rho \cdot n_{flask}^3 \cdot d_{flask}^4 \cdot \sqrt[3]{V_{flask}}} \quad (6)$$

**Shake flask**		
Density	ρ	1000 kg m^{-3}
Shaking frequency	n_{flask}	3.5 s^{-1}
Flask diameter	d_{flask}	0.085 m
Dynamic viscosity	η	0.001 Pa s (assumption)
Filling volume	V_{flask}	0.0004 m^3
Reynold number	Re	25287.5
Modified Power number	Po'_{flask}	0.257284464
Absolut power input	$P_{flask,aerated}$	0.019693069 W
Volumetric power input	$\frac{P_{flask,aerated}}{V_{flask}}$	0.492326717 W L^{-1}

As scale up criteria, the volumetric power input was chosen, therefore:

$$\frac{P_{flask,aerated}}{V_{flask}} = \frac{P_{reactor,aerated}}{V_{reactor}} \quad (7)$$

$$\frac{P_{reactor,aerated}}{P_{reactor,unaerated}} = \left(1.384 + \left(735 \cdot \frac{u_g}{\sqrt{g \cdot D_R}}\right)^2\right)^{-0.5} + 0.15 \quad (8)$$

$$u_g = \frac{q_g \cdot V_{reactor}}{A_{reactor}} \qquad (9)$$

$$P_{reactor,unaerated} = Po_{reactor} \cdot \rho \cdot n_{stirrer}^3 \cdot d_{stirrer}^5 \qquad (10)$$

**Stirred tank reactor and results of equations**		
Volumetric power input	$\frac{P_{flask,aerated}}{V_{flask}} = \frac{P_{reactor,aerated}}{V_{reactor}}$	0.492326717 W L^{-1}
Absolut power input (aerated)	$P_{reactor,aerated}$	1.476980151 W
Reactor diameter	D_R	0.16 m
Gravity constant	g	9.81 m s^{-1}
Stirrer diameter	$d_{stirrer}$	0.064 m
Filling volume	$V_{reactor}$	0.003 m^3
Gas-liquid surface	$A_{reactor}$	0.06310144 m^2
Aeration rate	q_g	0.0000125 m^3 s^{-1}
Gas velocity	u_g	1.98E-04 m s^{-1}
Absolut power input (unaerated)	$P_{reactor,unaerated}$	1.944857117 W
Power number (unaerated!)	$Po_{reactor}$	4.9
**Stirring rate**	$\boldsymbol{n_{stirrer}}$	**7.176795701 s^{-1} → 430 rpm**

2 All mycofactocin congeners observed in shake flask cultivations

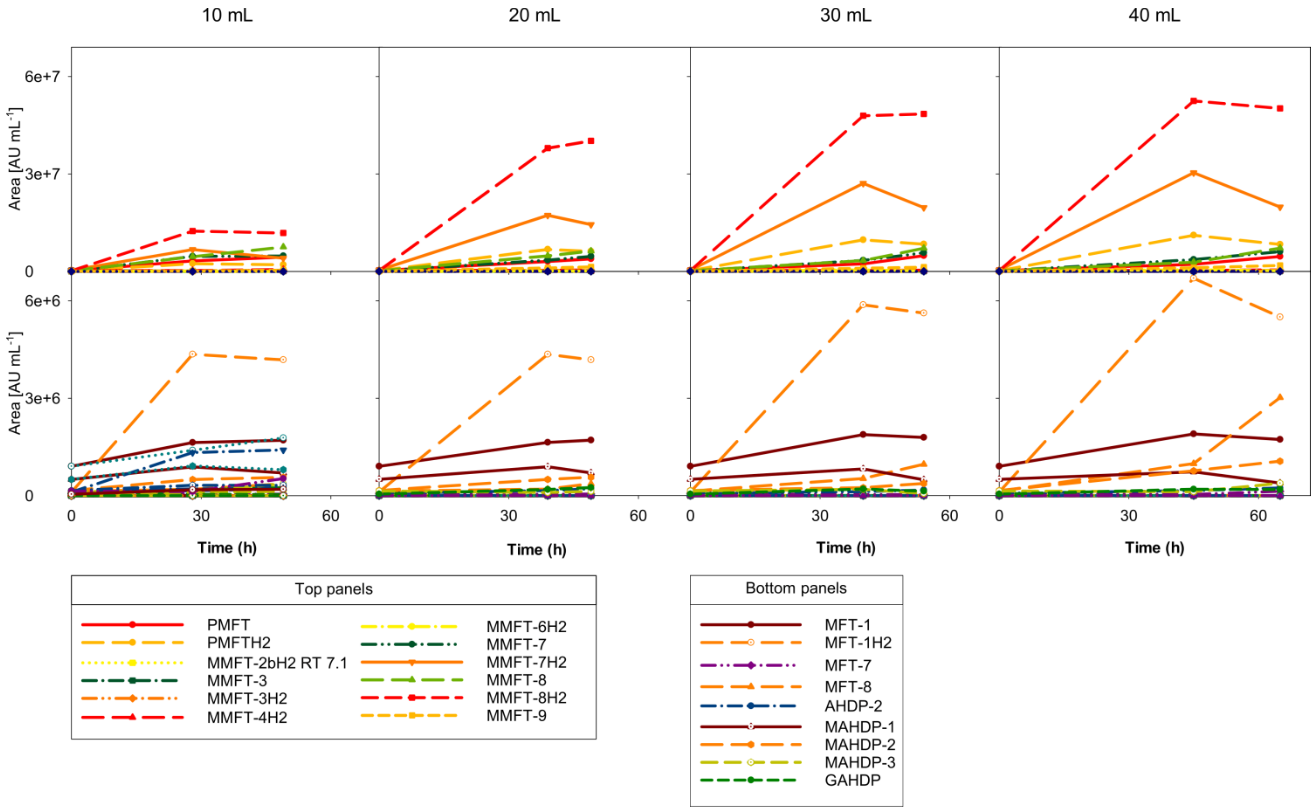

Figure S1 All mycofactocin congeners observed in shake flask TOM device. The oxygen transfer was varied by different filling volumes of the shake flasks. Panel **(A)** 10 mL; **(B)** 20 mL; **(C)** 30 mL; **(D)** 40 mL. Experimental conditions: Shaking diameter = 25 mm, shaking frequency = 210 rpm, total flask volume = 250 mL, filling volume = 10 to 40 mL, temperature = 37°C, LB medium with 10 g L^{-1} ethanol, 250 mM MOPS buffer, initial pH = 7.2.

3 Strongly oxygen-limited batch culture of *M. smegmatis* in a 7 L stirred tank reactor

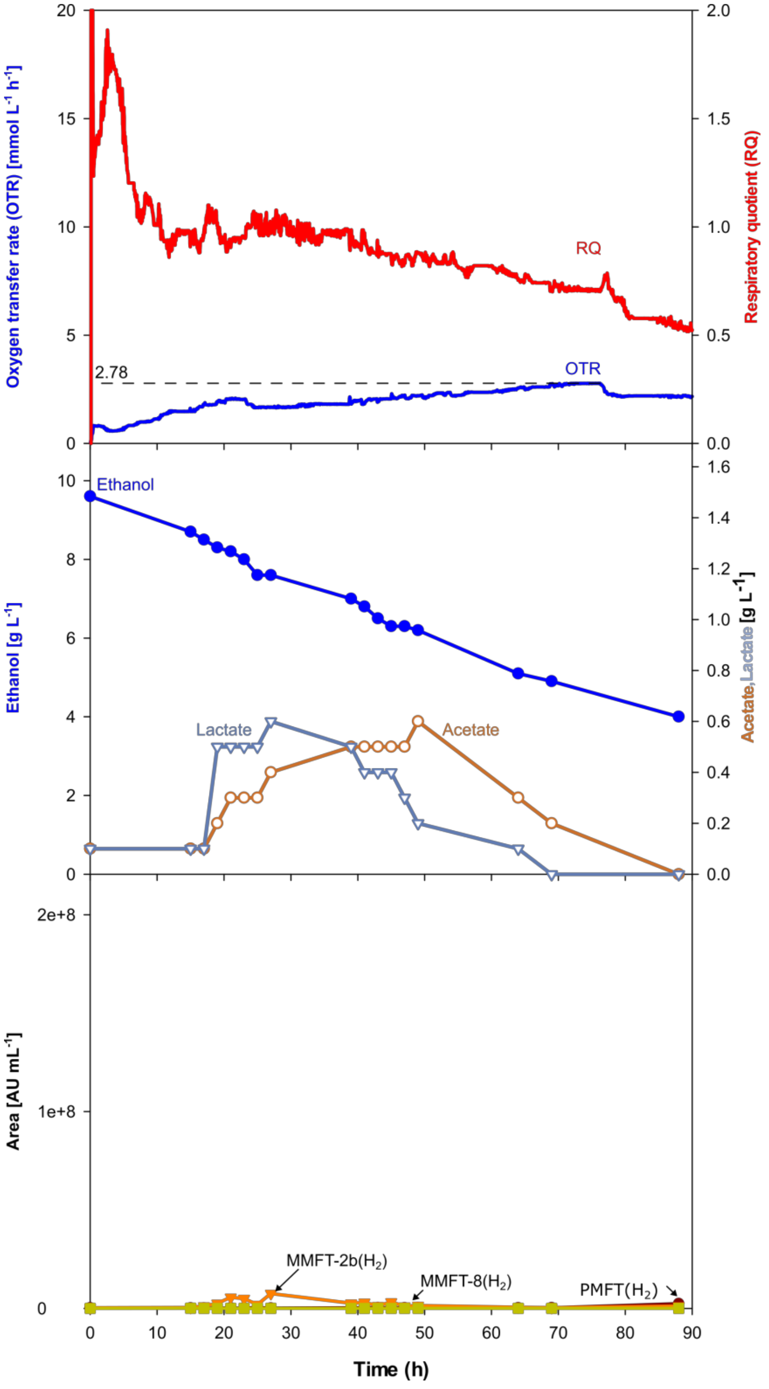

Figure S2 Strongly oxygen-limited batch culture of *M. smegmatis* mc2 155 in a 7 L stirred tank reactor. (A) Oxygen transfer rate (blue) and respiratory quotient (red); **(B)** offline metabolites ethanol (◇), acetate (△), lactate (◇); **(C)** peak area of selected mycofactocin species by LC-MS. PMFT (●), MMFT-2b (△), MMFT-8 (▼). Experimental conditions: Stirring rate = 150 rpm, gas flow rate = 0.75 L min^{-1} (= 0.25 vvm), total reactor volume = 7 L, filling volume = 3 L, temperature = 37°C, LB medium with 10 g L^{-1} ethanol.

4 All mycofactocin congeners found in batch culture of *M. smegmatis* mc2 155 in 7 L stirred tank reactors.

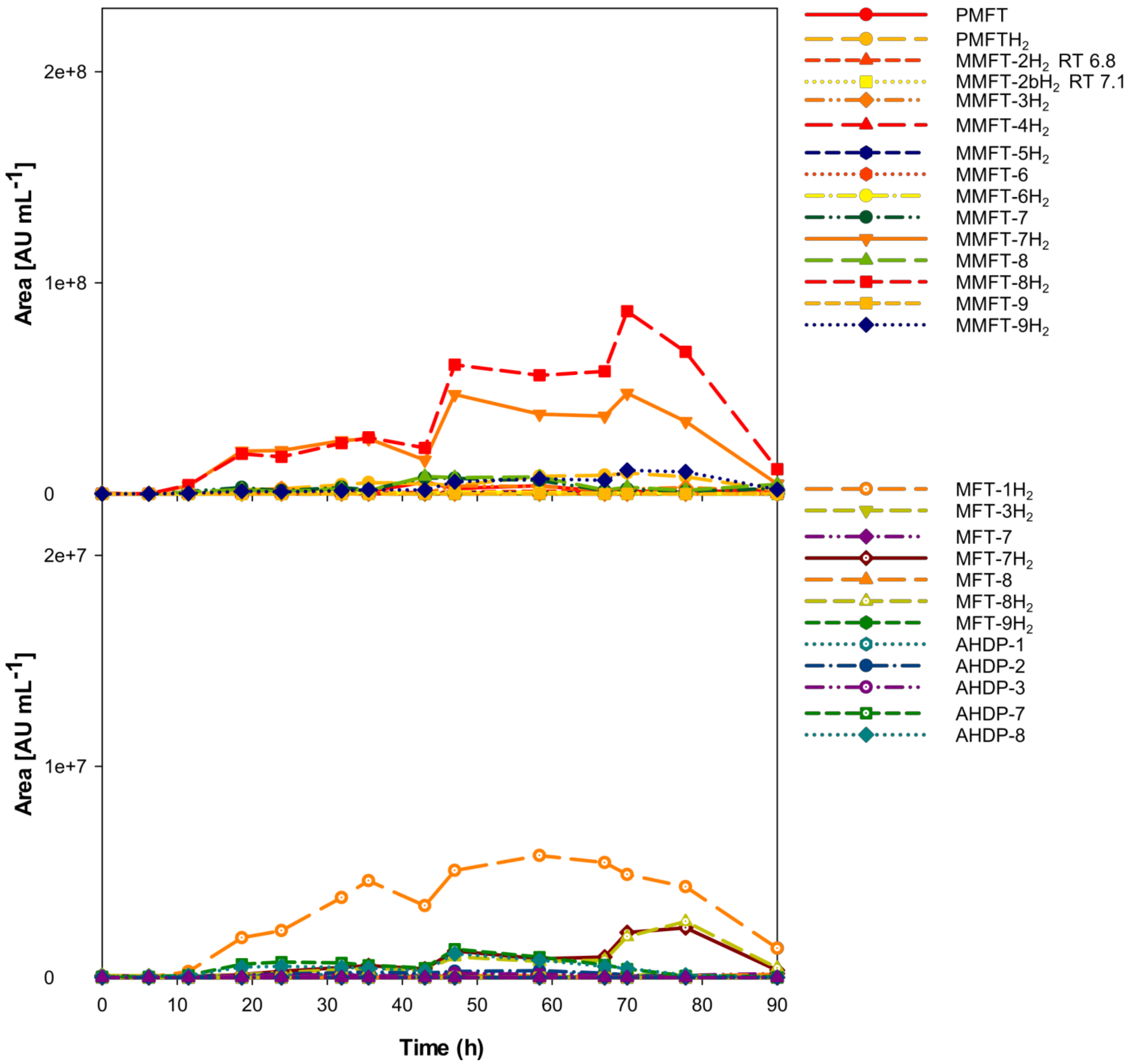

Figure S3 All mycofactocin congeners found in batch culture of *M. smegmatis* mc^2 155 in 7 L stirred tank reactors under oxygen-limited conditions in complex media.

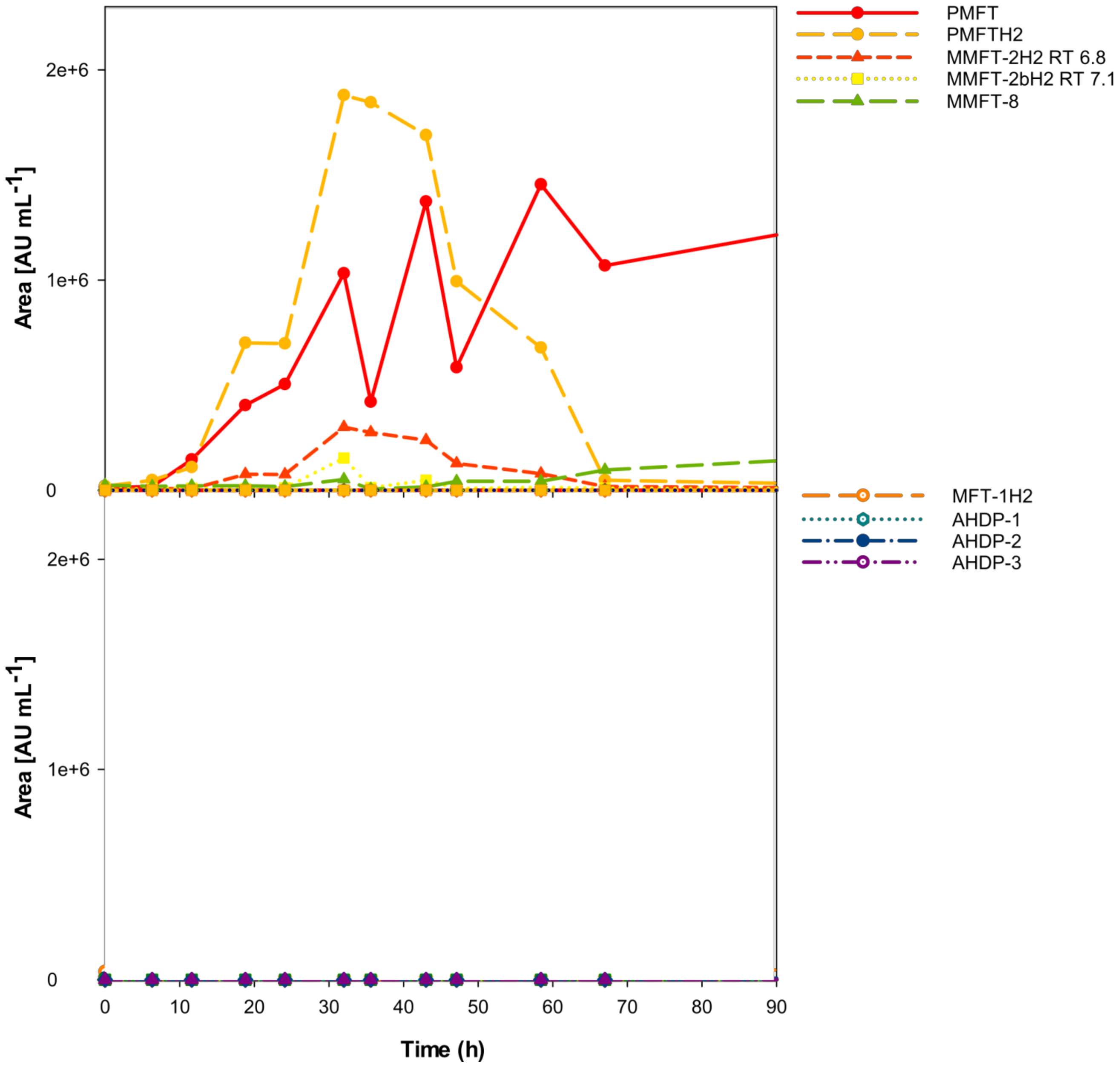

Figure S4 All mycofactocin congeners found in batch culture of *M. smegmatis* mc^2 155 in 7 L stirred tank reactors under oxygen-unlimited conditions in complex media.

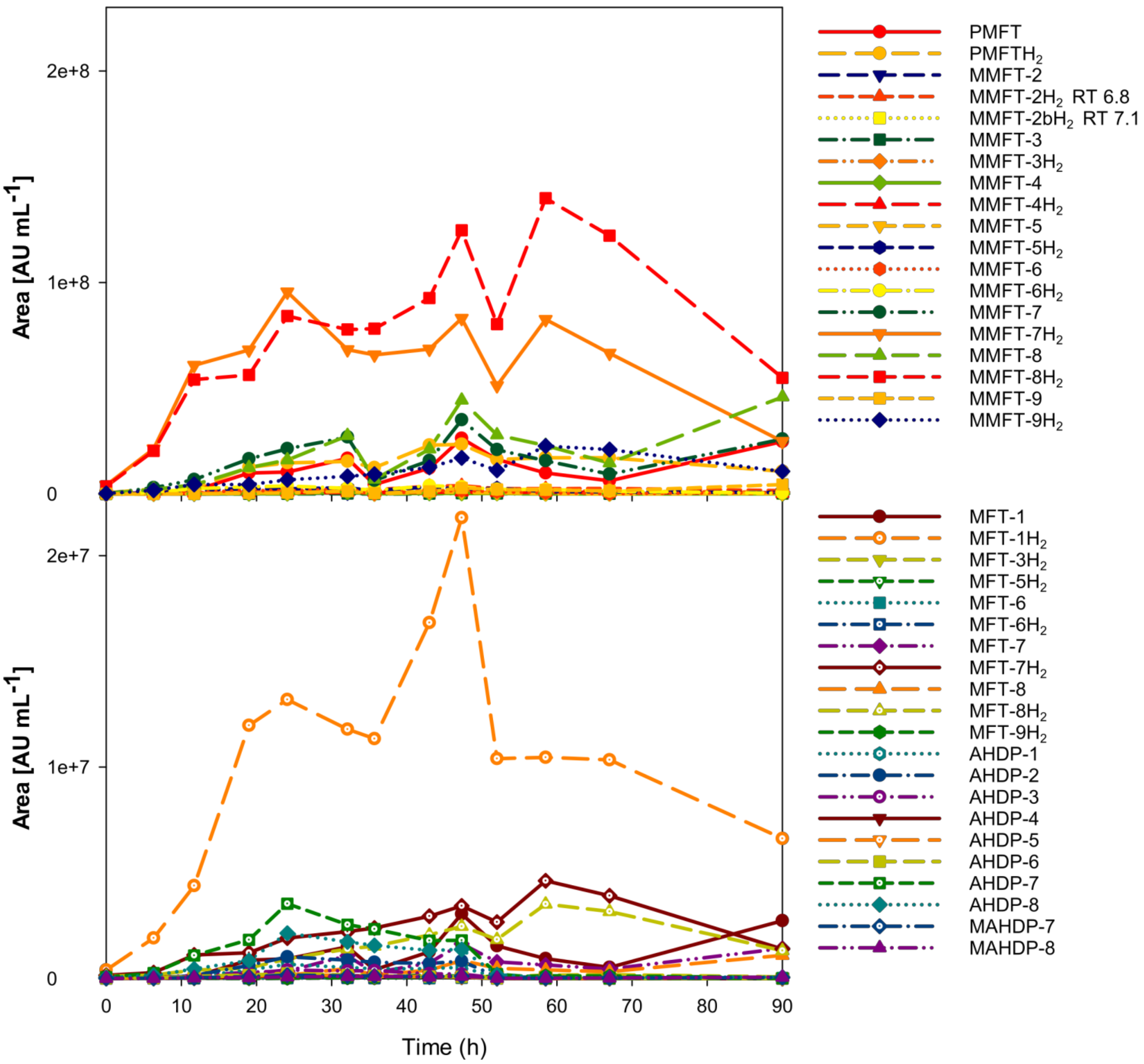

Figure S5 All mycofactocin congeners found in batch culture of *M. smegmatis* mc^2 155 in 7 L stirred tank reactors under oxygen-limited conditions in mineral media.

5 Growth at the reactor wall

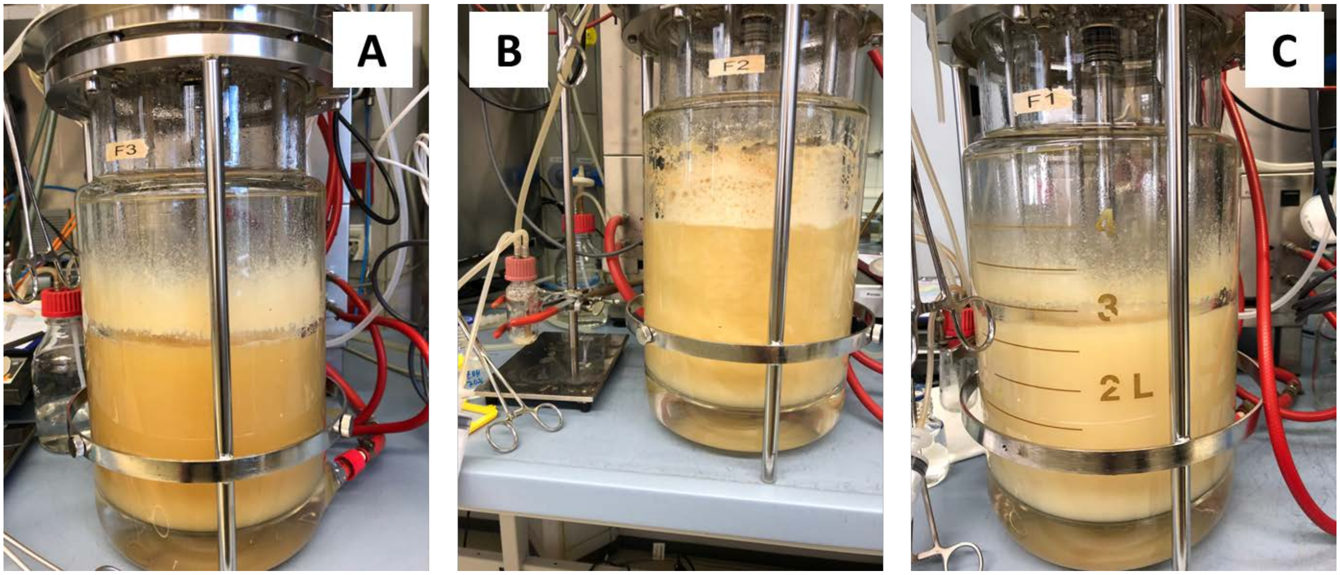

Figure S6 Foam formation and growth at the reactor wall. **(A)** Complex medium, oxygen-limited conditions (see Fig. 3); **(B)** Complex medium, oxygen unlimited conditions (see Fig. 4); **(C)** Mineral medium, oxygen-limited conditions (see Fig. 5)

6 Additional information for LC-MS analysis:

Name		Formula	Monoisotopic mass (calculated)	RT (min)
Premycofactocin precursors	PMFT	$C_{13} H_{15} N O_3$	233.10519	7.17
	$PMFTH_2$	$C_{13} H_{17} N O_3$	235.12084	6.83
Mycofactocinones	MFT-1	$C_{19} H_{25} N O_8$	395.15802	7.16
	MFT-7	$C_{55} H_{85} N O_{38}$	1367.47497	6.96
	MFT-8	$C_{61} H_{95} N O_{43}$	1529.5278	6.86
Mycofactocinols	MFT-1H_2	$C_{19} H_{27} N O_8$	397.17367	6.82
	MFT-3H_2	$C_{31} H_{47} N O_{18}$	721.27932	6.53
	MFT-7H_2	$C_{55} H_{87} N O_{38}$	1369.49062	6.54
	MFT-8H_2	$C_{61} H_{97} N O_{43}$	1531.54345	6.51
	MFT-9H_2	$C_{67} H_{107} N O_{48}$	1693.59627	6.48
Methyl mycofactocinones	MMFT-6	$C_{50} H_{77} N O_{33}$	1219.4378	7.25
	MMFT-7	$C_{56} H_{87} N O_{38}$	1381.49062	7.26
	MMFT-8	$C_{62} H_{97} N O_{43}$	1543.54345	7.15

	MMFT-9	$C_{68} H_{107} N O_{48}$	1705.59627	7.11
Methyl mycofactocinols	MMFT-2H_2	$C_{26} H_{39} N O_{13}$	573.24215	6.83
	MMFT-3H_2	$C_{32} H_{49} N O_{18}$	735.29497	6.81
	MMFT-4H_2	$C_{38} H_{59} N O_{23}$	897.3478	6.83
	MMFT-5H_2	$C_{44} H_{69} N O_{28}$	1059.40062	6.82
	MMFT-6H_2	$C_{50} H_{79} N O_{33}$	1221.45345	6.88
	MMFT-7H_2	$C_{56} H_{89} N O_{38}$	1383.50627	6.83
	MMFT-8H_2	$C_{62} H_{99} N O_{43}$	1545.5591	6.80
	MMFT-9H_2	$C_{68} H_{109} N O_{48}$	1707.61192	6.76
Non-redox active	AHDP-1	$C_{19} H_{28} N_2 O_7$	396.18965	5.60
	AHDP-2	$C_{25} H_{38} N_2 O_{12}$	558.24248	5.60
	AHDP-3	$C_{31} H_{48} N_2 O_{17}$	720.2953	5.60
	AHDP-7	$C_{55} H_{88} N_2 O_{37}$	1368.50661	5.60
	AHDP-8	$C_{61} H_{98} N_2 O_{42}$	1530.55943	5.60
	MAHDP-1	$C_{20} H_{30} N_2 O_7$	410.2053	5.29
	MAHDP-2	$C_{26} H_{40} N_2 O_{12}$	572.25813	5.80
	MAHDP-3	$C_{32} H_{50} N_2 O_{17}$	734.31095	5.91
	MAHDP-8	$C_{62} H_{100} N_2 O_{42}$	1544.57508	5.79
	MAHDP-9	$C_{68} H_{110} N_2 O_{47}$	1706.62791	5.78
	GAHDP	$C_{15} H_{21} N_3 O_3$	291.15829	5.68
